# Physiological Profiling of Agitation in Dementia: Insights From Wearable Sensor Data

**DOI:** 10.1093/geroni/igae057

**Published:** 2024-06-05

**Authors:** Hannah Davidoff, Alex Van Kraaij, Laura Van den Bulcke, Erika Lutin, Mathieu Vandenbulcke, Nick Van Helleputte, Maarten De Vos, Chris Van Hoof, Maarten Van Den Bossche

**Affiliations:** Department of Electrical Engineering, ESAT, KU Leuven, Heverlee, Belgium; Imec, Heverlee, Belgium; OnePlanet Research Center, Wageningen, Netherlands; Geriatric Psychiatry, University Psychiatric Center KU Leuven, Leuven, Belgium; Neuropsychiatry, Research Group Psychiatry, Department of Neurosciences, Leuven Brain Institute, KU Leuven, Leuven, Belgium; Imec, Heverlee, Belgium; Geriatric Psychiatry, University Psychiatric Center KU Leuven, Leuven, Belgium; Neuropsychiatry, Research Group Psychiatry, Department of Neurosciences, Leuven Brain Institute, KU Leuven, Leuven, Belgium; Imec, Heverlee, Belgium; Department of Electrical Engineering, ESAT, KU Leuven, Heverlee, Belgium; Department of Development and Regeneration, Faculty of Medicine, KU Leuven, Leuven, Belgium; Department of Electrical Engineering, ESAT, KU Leuven, Heverlee, Belgium; OnePlanet Research Center, Wageningen, Netherlands; Geriatric Psychiatry, University Psychiatric Center KU Leuven, Leuven, Belgium; Neuropsychiatry, Research Group Psychiatry, Department of Neurosciences, Leuven Brain Institute, KU Leuven, Leuven, Belgium

**Keywords:** Agitation detection, Agitation physiology, Autonomic nervous system, Personalized models, Wearable sensors

## Abstract

**Background and Objectives:**

The number of people with dementia is expected to triple to 152 million in 2050, with 90% having accompanying behavioral and psychological symptoms (BPSD). Agitation is among the most critical BPSD and can lead to decreased quality of life for people with dementia and their caregivers. This study aims to explore objective quantification of agitation in people with dementia by analyzing the relationships between physiological and movement data from wearables and observational measures of agitation.

**Research Design and Methods:**

The data presented here is from 30 people with dementia, each included for 1 week, collected following our previously published multimodal data collection protocol. This observational protocol has a cross-sectional repeated measures design, encompassing data from both wearable and fixed sensors. Generalized linear mixed models were used to quantify the relationship between data from different wearable sensor modalities and agitation, as well as motor and verbal agitation specifically.

**Results:**

Several features from wearable data are significantly associated with agitation, at least the *p* < .05 level (absolute β: 0.224-0.753). Additionally, different features are informative depending on the agitation type or the patient the data were collected from. Adding context with key confounding variables (time of day, movement, and temperature) allows for a clearer interpretation of feature differences when a person with dementia is agitated.

**Discussion and Implications:**

The features shown to be significantly different, across the study population, suggest possible autonomic nervous system activation when agitated. Differences when splitting the data by agitation type point toward a need for future detection models to tailor to the primary type of agitation expressed. Finally, patient-specific differences in features indicate a need for patient- or group-level model personalization. The findings reported in this study both reinforce and add to the fundamental understanding of and can be used to drive the objective quantification of agitation.

**Translational Significance:** Agitation, a critical symptom in dementia, is typically measured through observation and psychological scales, introducing observer bias. This study presents results building the foundation for objective quantification of agitation, thus improving the understanding of patient responses, and facilitating future tailored detection models. These models can subsequently be used to alert the need for intervention therefore decreasing agitation frequency, enhancing intervention monitoring, and ultimately improving the quality of life of both people with dementia and their caregivers.

In our aging population, the number of people with dementia is expected to triple to 152 million in 2050 (Dementia Statistics | Alzheimer’s Disease International (ADI), n.d.). On top of the typical loss of cognitive functioning in dementia, behavioral, and psychological symptoms of dementia (BPSD), affecting up to 90% of all people with dementia, are clinically relevant (Cerejeira et al., 2012). One of the most critical BPSDs, agitation, as defined by the International Psychogeriatric Association (Cummings et al., 2014), encompasses behaviors across multiple categories associated with emotional distress: excessive motor activity, verbal aggression, and physical aggression. People with dementia and accompanying BPSDs experience more rapid cognitive deterioration and worsening of dementia symptoms (Canevelli et al., 2013; Ismail et al., 2016), reduced well-being and life satisfaction (Karttunen et al., 2011; Tatsumi et al., 2009), increased strain and stress for their caregivers (Chen et al., 2018; Ryu et al., 2011), and higher healthcare expenses (Costa et al., 2018).

The management of agitation, whether through nonpharmacological or pharmacological interventions, often relies on subjective and incomplete observations by caregivers. Due to the disruptive nature of agitated behavior and limited comprehension of its root causes, agitation can drive the (over)use of pharmacological interventions despite high morbidity and mortality of these medications in older adults (Salzman et al., 2008; Selbæk et al., 2008). There is a need to monitor agitation directly and objectively to further understanding of patient response; eventually enabling the identification of physiological markers of agitation.

Some theoretical basis for a patient’s agitation response has been provided by unimodal studies researching the impact of a single factor and its relationship to agitation. Relationships between increased movement with agitation (Knuff et al., 2019; Nagels et al., 2006; Valembois et al., 2015) on an hourly-/daily-/weekly-level have been identified, in contrast to the momentary level used here. Additionally, increases in baseline skin conductance levels (SCL; derived from electrodermal activity [EDA] sensors) are correlated with agitation (Kikhia et al., 2016; Melander et al., 2017). Features like these, derived from EDA activity, have been shown to reflect autonomic nervous system (ANS) activation (also known as a stress or arousal response; Boucsein, 2012). Other physiological parameters regulated by the ANS, such as heart rate (HR) and skin temperature (ST), have not yet been explicitly associated with agitation individually. These two modalities have been combined with other wearable-derived data in multimodal research focusing on using machine learning to develop agitation detection models (Au‐Yeung et al., 2020; Bankole et al., 2020; Iaboni et al., 2022). These models have shown promise in understanding the complexities of agitation response, both as a whole and when broken down into individual feature components.

However, a more comprehensive understanding of agitation necessitates an additional focus on different types of agitation expressed. The majority of research focused on responses when a specific kind of agitation is expressed, focused on verbal agitation, and linked verbal agitation to either pain, physiological arousal, or other kinds of discomfort (Cohen-Mansfield et al., 1992; Husebo et al., 2022; Kim & Park, 2018). Stress, a concept closely tied to arousal (Tutunji et al., 2023; Winsky-Sommerer et al., 2005), has been related to behavioral symptoms in people with dementia in the Progressively Lowered Stress Threshold model (Gerdner et al., 2005; Hall & Buckwalter, 1987; Richards & Beck, 2004). This model hypothesizes that people with dementia have a lowered stress threshold affecting their ability to cope with daily stressors, and that this threshold decreases throughout each day. Therefore, this model associating agitation with stress, combined with the results from the studies of Melander and Kikhia associating agitation with a state of physiological arousal (Kikhia et al., 2016; Melander et al., 2017), establishes the basis for the analysis presented here. In this study, an exploratory analysis is presented of how physiological and movement parameters from a wrist-worn wearable, the Chill+, relate to a patient’s agitation response and then expand upon this analysis by looking at how the response differs by specific type of agitation expressed or by patient. This study is the first data-driven research stemming from the data collection protocol described in Davidoff et al. (2022) with the objective of providing a more all-encompassing view of informative features for the detection of agitation.

The main research question for this study is which features of the wristwatch wearable, the Chill+, if any, are informative for detecting agitation.

More specifically the following questions are discussed:

(1) What does a patient’s physiological response look like when agitated compared to their nonagitated state?
(2) Are there different response profiles for different agitation types (motor vs verbal)?
(3) Is there high variability in patient-specific response profiles when agitated?

The objectives of this paper align with the first overall study objective, related to agitation response, mentioned in the protocol and study design paper (Davidoff et al., 2022). The results presented here will provide the foundation for future work in both agitation detection models and subsequent prediction of agitation when considering context, such as environment/location/sleep.

## Method

The data collection protocol used follows a cross-sectional repeated measures design. Further detailed information regarding the recruitment and data collection protocol can be found in (Davidoff et al., 2022).

### Recruitment

Patients included in this study were recruited from a specialized neuropsychiatric ward for people with dementia and accompanying severe behavioral problems. This is also where the data collection, integrated into the day-to-day clinical workflow and approved by the Ethics Committee Research of University Hospitals Leuven (ID: S62882), took place. Stays on this ward have an average length of 6–8 weeks. The patients were recruited from a convenience sample.

### Data Collection

The data presented in this study results from a multimodal sensing study encompassing data from both wearable and fixed sensors with the goal of detecting agitation in people with dementia.

In this study, the focus is on the physiology at the moment of agitation, for which physiological data from the Chill+ wristwatch is used, as well as data from the Pittsburgh Agitation Scale (PAS), Richmond Agitation Sedation Scale (RASS), and room temperature data from the MetaTracker and MetaMotion (both sensors from MbientLab). Following is a short introduction to all the data collection instruments that are of relevance to this study.

#### Chill+

The Chill+ is put on the patient every included day after routine care and taken off before the patient goes to bed. This aligns roughly with the hours the surveys are filled in, although sleep-wake times vary per patient. This device measures both physiological parameters (photoplethysmography [PPG], EDA, and ST) and movement parameters with an accelerometer (ACC). The Chill+ also includes a gyroscope; this modality was left out of the analysis due to the limited interpretability of the basic features of this signal.

#### Metatracker/metamotion

Additionally, due to the influence of environmental temperature on EDA and ST values, the average ambient temperature across living spaces on the ward was also extracted from the Metatracker or Metamotion (mounted on sensor enclosures throughout the ward) data when surveys were filled in.

#### Agitation annotation—survey

The annotation of agitation is done by nurses on the ward using a study-specific app integrating the Experience Sampling Methodology to minimize recall bias (Myin‐Germeys et al., 2018). This annotation can happen both prompted by a signal at nine pseudorandom time points per day, as well as spontaneously with the observation of agitation. These time points are generated between 08 h 45 min and 20 h 45 min each day of inclusion, with a minimum spread of 50 min. In addition to the presence or absence of agitation, the psychological scales used within the surveys (PAS and RASS) also give information as to the severity and subtype of agitation, when present. These surveys were selected for their ease-of-use and good interrater reliability/correlations (Rosen et al., 1995; Sessler et al., 2002). Duplicate surveys, defined as occurring within 5 min of the previous, are removed from subsequent analysis. The physiological and movement data is then aligned by timestamp with the survey data measuring the patient’s agitation level multiple times a day.

#### Window of interest

For each survey filled in by the nurses on the ward, physiological, movement, and temperature data were extracted in a 6-min window, centered on the timestamp of survey submission. This 6-min window was the median duration of agitation across all agitation episodes recorded in the study by Iaboni et al. (2022). Given that this 6-min window overlaps with the occurrence of agitation, data from this window is considered part of the patients’ (physiologic and movement) response when agitated.

### Preprocessing and Quality Control for Wristwatch Data

Data pipelines were constructed per modality of the Chill+ data. For every 6-min window around the survey, data from each modality were extracted separately.

#### Accelerometer

For the ACC, this is the raw data sampled at 32 Hz. From the raw data in each of the cardinal directions, the magnitude of acceleration was calculated according to the formula:


$$\begin{array}{l} Acceleration=\sqrt{x^{2}+y^{2}+z^{2}} \end{array}$$

#### Photoplethysmogram

For the PPG sensor, the data extracted is the calculated HR as determined by a series of algorithms (both in the time and frequency domain) adapted from literature (Fedjajevs et al., 2020; Temko, 2017). When the application of these algorithms subsequently indicates low-quality data, the HR value is reported as NA. The resulting time series had a frequency of 0.5 Hz. Missing data was filled with a rolling 10 s median window, provided that the gap of missing data was less than the length of the rolling window. Remaining missing data points were then interpolated using default 1D spline interpolation (*k* = 3) when there is data present in the surrounding time points. After these preprocessing steps, when the proportion of missing values (also the number of values that would need imputing) per 6-min window is ≥50%, the window was dropped from subsequent analysis.

#### Electrodermal activity

For the EDA data, originally sampled at 256 Hz, the data is first filtered with a Savitzky–Golay filter to mitigate quantization noise (Thammasan et al., 2020) before downsampling to 8 Hz. The EDA data quality is calculated on this filtered 8 Hz signal using a random forest model that is pretrained on an independent ambulatory EDA data set (Pattyn et al., 2023). The 6-min windows with a quality indicator value of below 0.5 are removed. For the windows with sufficient quality, the artifacts are removed and interpolated as described in Pattyn et al. (2023). After artifact removal and interpolation, the signal is decomposed into the phasic and tonic components, using the approach of Ledapy (https://github.com/HIIT/Ledapy), which is a Python (https://python.org) implementation of LedaLab (Benedek & Kaernbach, 2010) that relies on a deconvolution approach. The phasic component, or skin conductance response, is the fast-changing part of the EDA signal representing the sympathetic activation of sweat glands in the wrist (ANS activation) (Boucsein et al., 2012; Regalia et al., 2023). The tonic component, or SCL, is made up of the slow-changing part of the EDA signal thought to represent an interaction between sympathetic activation of sweat glands and ST/hydration (Boucsein et al., 2012; Regalia et al., 2023).

#### Skin temperature

The ST data, sampled at 1 Hz, is checked to see if it falls within the range of 20–40°C (Jones & Lederman, 2006; Smets et al., 2018). If the proportion of data present and within this range is less than or equal to 50% the window is dropped from subsequent analysis.

#### Feature extraction for all signal modalities

For each of the four sensor modalities analyzed in this study, a minimal set of features was calculated using the Python time series feature extraction package: *tsfresh* (Christ et al., 2018). In order to summarize the signal and preserve interpretability, basic statistical features, such as the minimum, maximum, median, and standard deviation were calculated for each signal’s 6-min window surrounding the timestamp of survey completion. For the ST, an additional feature, the slope, was calculated to capture the change within the window of this slow-varying physiological signal. Additionally, all features were centered and scaled within the patient.

### Preprocessing for Agitation Surveys

Patients were removed from analysis if only one survey was present with high-quality wristband data for that modality. Additional reasons for excluding patient data from subsequent analysis include: incomplete study inclusion, sensor data loss, insufficient high-quality data (likely due to lack of acceptance of wearables), or inclusions when the protocol was being piloted and adjusted accordingly to capture relevant information. Given that patients included in each modality’s modeling differed, Table 1 shows any differences in demographics seen in subgroups. For the ACC and ST analyses, no patients were excluded, therefore the demographics for these modalities are equal to the demographics for the study population.

**Table 1.** Overall Patient Demographics, Split by Modality

Variable	Group		
	Study population (*n* = 30)	Patients included in analysis of EDA data (*n* = 25)	Patients included in analysis of HR data (*n* = 26)
Sex, *n*			
Women	10	9	9
Men	20	16	17
Age (years), M ± *SD*	80.4 ± 7.69	79.5 ± 7.43	80.8 ± 7.89
Diagnosis, *n*			
Alzheimer’s disease (AD)	11	10	9
Vascular (VaD)	3	2	2
Mixed (AD and VaD)	4	3	3
With Lewy bodies	3	2	2
Multiple etiologies	3	3	3
Other (due to another medical condition)	1	1	1
Not otherwise specified: 5	5	4	5
Time since diagnosis (years), M ± *SD*	3.2 ± 1.96	3.34 ± 2.15	3.11 ± 1.83
MMSE (# NAN = 2), M ± *SD*	13.5 ± 6.56	12.4 ± 6.44	13.5 ± 6.32
CMAI, M ± *SD*	55.4 ± 14.9	56.6 ± 16.0	55.3 ± 15.4
NPI (FxE), M ± *SD*	29.1 ± 14.2	30.6 ± 14.6	28.3 ± 14.1
Cornell, M ± *SD*	9.13 ± 4.08	9.4 ± 4.27	8.88 ± 4.31

*Notes*: CMAI = Cohen–Mansfield Agitation Inventory; EDA = electrodermal activity; HR = heart rate; MMSE = Mini-Mental State Examination; NPI = Neuropsychiatric Inventory.

For the analyses of the data from the accelerometer (ACC) and skin temperature (ST) sensor, the demographics = the study population, as none of the patients are removed from those analyses.

Data availability (number of surveys with high-quality data) per modality as well as splits per agitation types and number of surveys per patient were examined and shown in Table 2 as well as Supplementary Table A3. For Figures 3 and 4, five patients were selected that had consistent data availability: at least two agitated and nonagitated surveys with high quality sensor data across all agitation types and all sensor modalities. The data from these five patients forms the subset used for visualization.

**Table 2.** Data Availability per Modality and Patient

Variable	Modality			
	EDA (*n* = 442)	HR (*n* = 342)	ST (*n* = 746)	ACC (*n* = 792)
# of patients included	25	26	30	30
Median number of surveys per patient	20	10	25.5	28
Min–Max surveys per patient	2–36	2–44	2–59	2–64
Median percentage of agitated surveys per patient? (IQR)	25.0% (29.3%)	8.7% (28.2%)	26.3% (30.8%)	22.3% (30.1%)
% motor	3.8% (14.3 %)	0 (6.1%)	7.3% (11.3%)	7.1% (11.6%)
% verbal	0 (4.5%)	0 (0)	0 (3.7%)	0 (3.4%)
% both	5.6% (14.3%)	0 (9.2%)	6.4% (14.3%)	6.2% (14.2%)

*Notes*: ACC = accelerometer; EDA = electrodermal activity; HR = heart rate; ST = skin temperature; IQR = Interquartile range.

*n* indicates the total number of surveys with high-quality data for that data modality

The breakdown per patient is further described in the lower rows of the table.

### Modeling and Statistical Analysis

The main purpose of this research is to determine which features of the wristwatch wearable, the Chill+, if any, can be utilized for detecting agitation. Additionally, we wanted to provide preliminary insights into feature consistency across patients. To quantify the relationship between agitation and each of the extracted features, generalized linear mixed models were used, using the *glmer* function in R (Bates et al., 2015). For these models, with agitation as the outcome variable, each of the features extracted from each of the four wearable signal modalities was used as an explanatory variable in a separate model and included a random intercept for each patient. Given the low number of surveys with high-quality data available per patient, random slopes were not used as it would overcomplicate the model structure and prevent model convergence. All remaining high-quality data per modality was used in its respective model. Additionally, all features were centered and scaled within patient.

**Figure 1.**
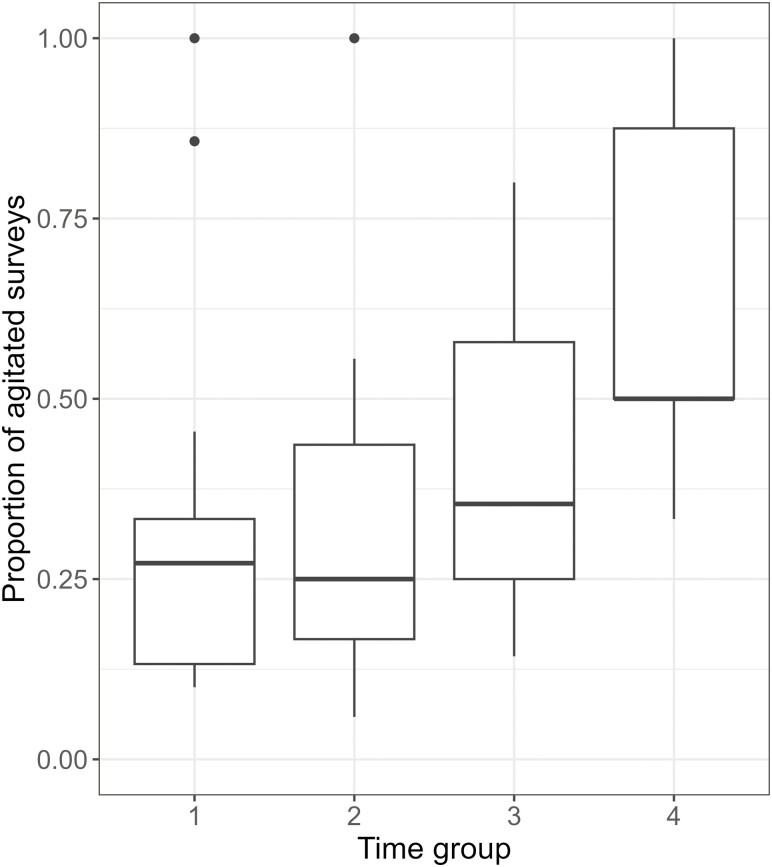
Proportion of agitated surveys per time group, calculated on a per patient level. Time group 1 includes the hours of 8–12 (exclusive), 2:12–16 (exclusive), 3:16–20 (exclusive), 4: 20–8 (exclusive).

The process of converting agitation into binary form was accomplished by assigning a value of 1 to the outcome if the overall score exceeded 0, thus indicating the presence of agitation. The same was done for motor and verbal agitation using the motor and verbal sub scores of the PAS respectively. Each of the models also included a time component (time block of the day, e.g., “time_group,” 1: 8–12 h, 2: 12–16 h, 3: 16–20 h, 4: 20–8 h) as a fixed effect, to account for the daily pattern of agitation (typical increases toward the late afternoon/evening due to sundowning (Volicer et al., 2001)). The proportion of agitated surveys per time group for each patient was calculated and shown in Figure 1. Additionally, we controlled for average environmental temperature in the models for EDA and ST features, and movement (using the standard deviation of acceleration) in the models for HR features, by including these variables in the respective models, as these factors may affect the physiological signals measured.

The details of the generalized linear mixed model structure for the agitation models can be found in Supplementary Material (A2). Three separate models were made for each feature extracted, one per outcome variable: agitation, motor agitation, and verbal agitation. For each of the three outcome variables and each of the features per sensor modality, the beta coefficients for the feature were reported along with their respective *p* values. These standardized coefficients can be interpreted as the effect size per feature as the explanatory variable and agitation (type) as the outcome variable (Nieminen, 2022). The *p* values are calculated with Wald tests, the default tests conducted by the *glmer* function, and were not corrected for multiple testing due to the exploratory nature of this analysis.

## Results

### Patient Demographics and Data Availability

Given the data availability per patient, the number of patients included in the statistical models differs from the total patient population included. The demographics shown in Table 1 summarize the patients included in each of the modality analyses. The demographics for the ACC and ST analyses are equivalent to the demographics of the complete study population (*n* = 30).

After the preprocessing and data quality filtering steps, the resulting data availability is shown in Table 2, split by data modality. Additional counts and percentages of surveys with high-quality data are shown in Supplementary Table A3. A 71.67% of surveys recorded had wearable data present. The percentage of surveys with high-quality data from the number with data present varies from 43.5% to 100% for HR and ACC modalities, respectively. Nonacceptance of the wearable is represented in 28.33% of surveys with no wearable data present. For the HR modality analysis, there is a lower number of surveys per patient, as shown by the median number of surveys per patient shown in Table 2.

Figure 1 shows an increasing proportion of agitated surveys by time group, suggesting an increase in the amount of agitation toward the end of the day. This underlies the importance of accounting for time of day, with the variable time_group, in the models.

### Informative Features: Population Level

**Figure 2.**
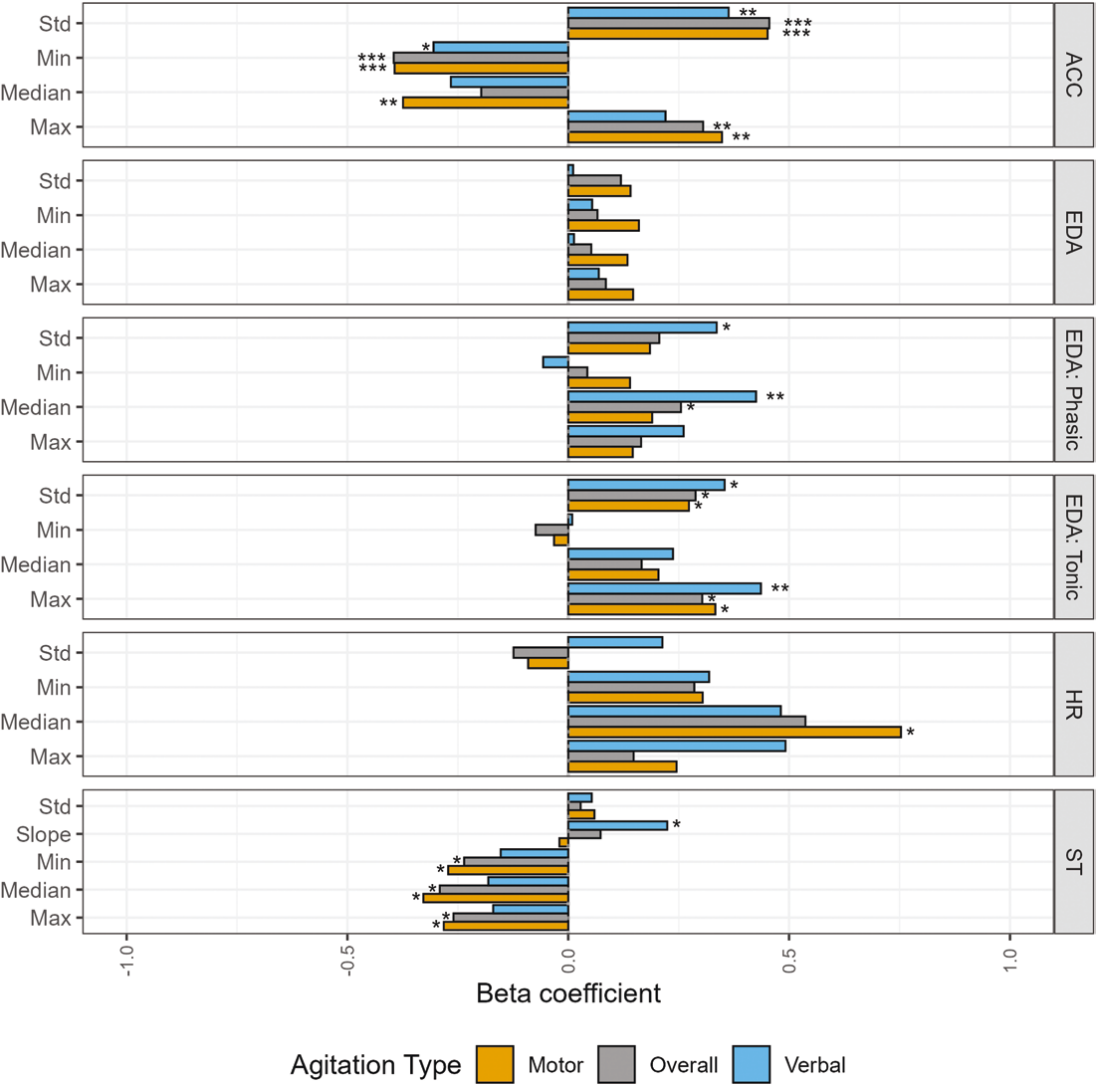
Beta coefficients (effect size) of each feature modeled using *glmer* models, colored by agitation type, grouped by sensor modality. Significance is shown with an asterisk. *p < .05, **p < .01, ***p < .001. ACC = accelerometer; EDA = electrodermal activity; HR = heart rate; *SD* = standard deviation; ST = skin temperature.

Figure 2 shows the estimated beta coefficients from the *glmer* models, using the data per modality shown in Table 2, separated by agitation type as the response variable. All coefficients visually represented here, as well as their *p* values, can be found in Supplementary Material A1. These coefficients can be interpreted as the change (increase or decrease) in the log-odds of the response variable, which in this case was the presence or absence of the different types of agitation, with a one standard deviation increase in the explanatory variable—the features listed on the y-axis, while the confounding variables are held constant. There are several physiological and movement related features that are significantly associated with agitation, either overall or motor/verbal specifically.

For the features derived from the ACC magnitude, the standard deviation, minimum, and maximum were found to be significantly associated with agitation overall (0.455: *p* < .001, −0.395: *p* < .001, and 0.305: *p* < .01, respectively). Additionally, the median ACC magnitude was significantly associated with motor agitation (−0.374: *p* < .01). Whereas, for verbal agitation as the response variable, only standard deviation and minimum had significant coefficients (0.363: *p* < .01 and −0.305: *p* < .05, respectively). It is important to note that the decrease in the minimum and increase in the maximum ACC magnitude here are both indicators of the range of the ACC magnitude around 1 g. Therefore, these two features can therefore both be interpreted as increased acceleration, while the standard deviation of acceleration can be interpreted as increased movement.

No features from the EDA signal, before decomposition into its tonic and phasic components, showed a significant association for any agitation response variables. However, after decomposition, several are significant explanatory variables when analyzing tonic and phasic features. From the tonic, or slow-changing, component of the signal, the standard deviation, as well as the maximum value, were significant for all types of agitation, albeit with differing significance levels (*p* < .05 for all except maximum tonic, which was *p* < .01 for verbal agitation, see Supplementary Material A1 for estimate values). The median level of the phasic (fast varying) component of the EDA signal was significant both overall (*p* < .05) and for verbal agitation (*p* < .01). Additionally, the standard deviation of the phasic component was only significantly associated with verbal agitation (*p* < .05). All EDA-related significant beta coefficients were positive, suggesting that an overall increase in EDA, or an increase in the change seen in EDA, indicates an increased probability of agitation.

Only median HR was significantly associated with motor agitation at the 0.05 level. A positive estimated coefficient of 0.736 suggests that an increase in HR above the intuitive increase with increased movement (controlled for in the model) would indicate an increased probability of agitation. Verbal agitation has a single explanatory variable with a trend toward significance: maximum HR, β = 0.492, *p* = .076.

Finally, when looking at ST features, three features significantly predict overall agitation and motor agitation: minimum, median, and maximum (−0.236 to −0.328). The negative sign of the estimated coefficients indicates that decreased ST is related to increased agitation. Interestingly, the slope of the ST has a positive coefficient estimate (0.224) and is significant at the 0.05 level for verbal agitation alone.

#### Informative features: split by agitation type or by patient

**Figure 3.**
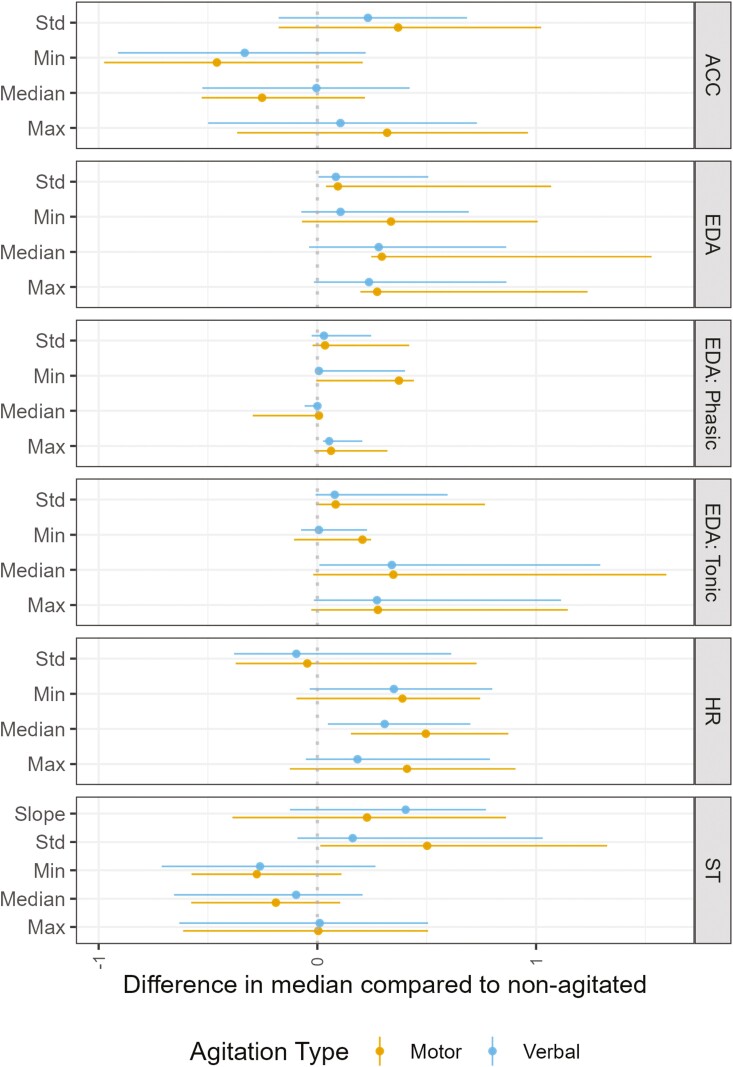
The difference in median standardized feature values of surveys with motor agitation/surveys with verbal agitation compared to non-agitated surveys. A difference of 1 would therefore indicate a 1 standard deviation difference in the standardized feature value of a motor or verbal agitation containing survey compared to a non-agitated survey. The dotted line at 0 is the reference standardized feature value of a “non-agitated” state. Std: standard deviation. ACC = accelerometer, EDA = electrodermal activity, HR = heart rate, ST = skin temperature.

Figure 3 shows the difference in the median standardized feature values for each of the different agitation types seen in surveys compared to their nonagitated counterparts. Differences plotted to the left of the reference (dotted line at 0), like three of the five ACC features as well as the standard deviation of the HR and four of the six ST features, indicate a standardized median feature value for either motor or verbal agitation that was less than the reference nonagitated (0) state. Differences plotted to the right of the reference (all remaining features) indicate a standardized median feature value for motor or verbal agitated surveys higher than the reference nonagitated state. Note that no significance tests were conducted between these groups in this way due to the inherent nesting/repeated measurements in the data, limited numbers of surveys in each group, and the exploratory nature of this analysis. Additionally, these median differences do not account for confounding factors, and summarize values across patients without weighting.

**Figure 4.**
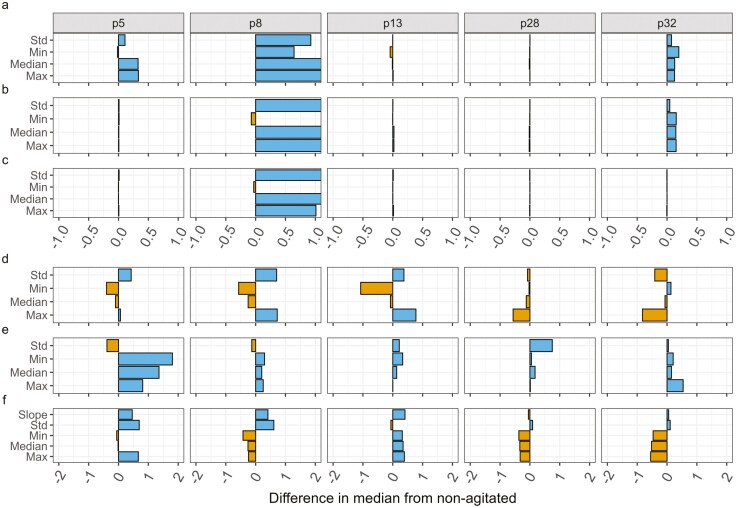
Median difference in standardized feature values between agitated and non-agitated surveys—grouped by patient. Note that this is a subset of patients chosen for data availability across sensor modality and agitation type. Further information on the criteria can be found in the “Preprocessing for agitation surveys” section. Model coefficient estimates take into account all available data per modality. (a) Electrodermal activity, (b) Electrodermal activity: phasic component, (c) Electrodermal activity: tonic component, (d) Accelerometer, (e) Heart rate, (f) Skin temperature.

Figure 4 shows patient-level differences in most informative features and differences in the direction of the effect seen when agitated. P8 shows large differences in EDA features when agitated (with a maximum difference in standardized medians of 3.36 for the median phasic feature), exceeding the axis limits (±1 standard deviation) set to enable visualization of smaller differences for the remaining four patients. These differences are relative to nonagitated surveys, meaning that large differences are the standard deviations above the standardized median value for nonagitated surveys.

In addition to the magnitude of the feature differences, Figure 4 shows the relative sign consistency across patients: most patients exhibit similar patterns of increase or decrease in certain features when agitated, compared to nonagitated surveys. For example, most patients show higher values (positive differences) of EDA and HR features, and lower values (negative differences) of ST features when agitated. However, there are some exceptions, such as P28, who shows small negative differences in EDA features while still having negative differences in ST features. Figure 4 does not show the split by agitation type, as the number of surveys of each type per patient is too small to draw meaningful conclusions. As with Figure 3, Figure 4 shows the standardized median feature differences without considering confounders that may increase or decrease the strength of a feature’s relationship with agitation. Population-level conclusions can be based on the results shown in Figure 1: the beta coefficient estimates taking into account patient-level differences as well as relevant confounding variables.

## Discussion

We presented analysis results based on wearable data from 30 people with dementia. Collectively, these results indicate that information derived from wearable sensors can be indicative of the presence of agitation. The results also demonstrated the need for group- or patient-specific models, with these models accounting for both agitation-type specific data as well as contextualizing confounding variables.

The importance of including time of day in a model for agitation is clarified in Figure 1, showing a clear increase in the proportion of agitation seen as the day progresses. This is expected due to consistent clinical observations of sundowning in this patient population (Volicer et al., 2001). The wearable compliance for this analysis, indicated by the percentage of surveys with wearable data shown in Supplementary Table A3 (71.67%), is in line with compliance in a similar population (Okhravi et al., 2023). The data quality seen per modality is also in line with wearable data quality from the Chill+ wristwatch in ambulatory studies (Krause et al., 2024). Overall, Figure 2 shows the amount of information each explanatory variable provides, split by agitation type and including all agitation. On a population level, several of these variables contribute a significant amount of information for the detection of agitation—and, therefore, for the development of digital biomarkers.

The ACC-derived features with significant beta coefficients match the increased movement often seen in agitation. The standard deviation of the magnitude of acceleration is used as a proxy for activity level due to being representative of these swings between deceleration and acceleration in various directions (Smets et al., 2018). Lower minimums and higher maximums of ACC magnitude are caused by abrupt changes in direction likely related to motor agitation. Knuff et al. (2019) showed the relationship between increased movement and agitation, where agitation was scored on a per patient level and not a moment-to-moment level like in this study. The increased movement with increased agitation relationship that we find here is in line with their findings and those from other actigraphic studies (Knuff et al., 2019; Nagels et al., 2006). Iaboni et al. (2022) found that ACC features contributed highly to agitation detection models across patients, specifically for physical aggression detection. This result is also in line with the result found here.

In this population, there was limited information in the EDA signal when the signal was not deconvolved. This is possibly due to the relevant signal being lost in the inherent noise and complexity of this signal modality. The loss of information is apparent when comparing the coefficient estimates of the complete EDA signal (largest coefficient estimate of 0.160) to estimates derived for features from the tonic and phasic components of the EDA signal (largest coefficient estimate of 0.436). These smaller coefficients combine with the lack of significant explanatory variables across any agitation type for the EDA features derived from the complete signal compared to the five significant explanatory variables out of the tonic and phasic component features.

The positive significant coefficient estimates seen for several tonic (slow-changing EDA) features in predicting agitation, motor agitation, and verbal agitation indicate an increase in baseline SCL, as well as changes in sympathetic nervous system arousal (indicated by the standard deviation; Boucsein et al., 2012). Similarly, the significant association of the phasic component indicates ANS activation due to an elevated median level with more frequent peaks (skin conductance responses). This result is consistent with the results reported in an EDA-specific study by Melander et al. (2017), which focused primarily on the tonic component of the skin conductance signal. Melander et al. (2017) also attribute their results to detecting stress responses before agitation. The larger, positive coefficient estimates and higher number of significant explanatory variables for the phasic and tonic features when predicting verbal agitation could indicate a more consistent state of arousal with verbal agitation compared to the limited number of significant EDA-derived variables for motor agitation. This hypothesis is in line with the current state of the art specifically focused on verbal agitation that has linked verbal agitation and other related vocalizations specifically to “physiological discomfort” or pain (Hoti et al., 2023; Husebo et al., 2022; Kim & Park, 2018). The difference in estimates when compared to motor agitation could also be due to additional factors, ranging from differences in care practices with this agitation type (e.g., differing medication administration or timing of care administration) to the quality of the resulting EDA signal when a patient is verbally agitated compared to motorically agitated. One could expect a decrease in EDA signal quality with motor agitation. Despite filtering out bad-quality EDA segments, it is likely that motor agitation segments are more affected by motion artifacts than their verbal agitation counterparts. Verbal agitation is considered one of the more difficult types of agitation to treat (Lemay & Landreville, 2010; McMinn & Draper, 2005). Subsequently, it may not respond to pharmacological intervention as readily as other types of agitation, leading to the eventual isolation of the verbally agitated patient (McMinn & Draper, 2005). The significant explanatory variables derived from the EDA components specifically for verbal agitation point toward a future possibility to quantify not only agitation stemming from discomfort but the discomfort itself; given the difficulties this patient population has when trying to express needs, this quantification alone could already aid in treatment plans.

The consistent positive coefficient estimates across all HR features, except standard deviation, suggest that an elevated HR could indicate an increased probability of agitation. This trend aligns with the probable ANS activation also seen in EDA and ST features. However, median HR was only significantly associated with motor agitation. This increase is also reflected in the HR section of Figure 2. The lower number of surveys per patient is likely a reason for limited significant features despite the large effect sizes shown in Figure 2. The HR models controlled for movement only during the 6-min window surrounding the survey. Therefore, HR may be elevated because of motor-agitated related movement just prior to the analyzed window, remaining elevated during the window despite decreases in movement. Maximum HR shows a trend toward significance for verbal agitation (*p* = .076). This feature has a larger coefficient estimate than median HR could indicate that the HR has already recovered after reaching a maximum value during the 6-min period, in contrast to a sustained increased HR that may be seen with motor agitation.

Furthermore, a smaller difference between HR for the presence and absence of verbal agitation could be due to a generally increased baseline HR for patients who typically express this type of agitation. This upward-shifted HR pattern could lead to a smaller relative change in HR when the patient becomes verbally agitated and, therefore, explains the lack of significance in this data set. The lack of significant heart rate-derived features for agitation overall, in contrast to EDA-derived features, points toward the EDA signal potentially being more informative across agitation types or that HR features are more likely to be influenced by factors not yet controlled for in the model. This difference could also be due to how these two physiological signals are controlled. Despite these differences, the consistent positive coefficient estimates across both signal modalities match the possible ANS activation seen in agitation.

Finally, the lower STs seen in overall agitation and motor agitation, reflected by the significant negative coefficient estimates for minimum, maximum, and median, are also in line with ANS activation. This lowering is expected as blood is drawn away from extremities, such as the hands/wrists, and core body temperature increases (Vinkers et al., 2013). Surprisingly, the slope of the ST, significant for verbal agitation alone, has a positive coefficient estimate. This trend could be due to, as also seen in the maximum HR, a recovery response after ANS activation, resulting in the return of blood flow to the extremities and a subsequent increase in distal ST.

The agitation-type split shown in Figure 3 makes it clear that, depending on the type of agitation primarily expressed, different features provide varying amounts of information. When the data are segmented in this manner, they reveal a perspective that differs from the aggregated agitation results on the population level. This is likely due to confounding variables (e.g., time of day, ambient temperature, and movement) not being included in the visualization. Differences in most informative features seen between motor and verbal agitation do not come across when these confounding variables are not accounted for. Despite the aggregation across patients and lack of inclusion of confounders in the agitation-type specific visualization (Figure 3), the expected general positive trend of EDA features is consistently present. Other population-level trends (seen in Figure 2), like increased movement represented by an increased standard deviation of ACC magnitude and decreases in minimum coinciding with increases in maximum (indicating increased range of movement), increased HR, and decreased ST are all replicated here.

The sign consistency is shown across patients in the patient-specific split visualization, Figure 4, matching the result found on a population level (Figure 2), can help identify common physiological markers of agitation. However, the magnitude of the difference can vary greatly depending on the patient and the feature. Patient-level differences could be explained by differences in demographics, such as age or diagnosis, that can influence the activation of the ANS (Gavazzeni et al., 2008); for example, patients with Lewy body dementia typically show symptoms of autonomic dysfunction (Palma & Kaufmann, 2018). Therefore, patient-specific or group-level personalized models may be more effective than generalized ones for detecting agitation.

The inclusion of the visualizations showing agitation-type and patient-level splits, Figures 3 and 4, further clarifies the need to include confounders—such as time of day, ambient temperature, or movement—as well as agitation types and patient specifics in agitation detection models due to the differences across features when splitting by these variables.

Overall trends across modalities suggest that agitation could be related to ANS activation, as reflected by significant increases in EDA features and decreases in ST features combined with HR elevation across agitation types. It may be possible to distinguish between agitation types using the response profiles of various physiological features. Finding informative features on a population level in this relatively small sample size indicates a high potential for driving future model development aiming to detect agitation. However, these models should be personalized on either a group or individual level taking into consideration more nuanced patient-level information.

Although the models in this study controlled for key confounding factors per sensor modality, more comprehensive models on a larger data set may allow for a deeper understanding of a patient’s response when agitated. Furthermore, due to the exploratory nature of our study, multiple testing was not corrected. Future hypothesis-driven work could reinforce these results. In our study protocol, it is difficult to know the exact timing of agitation onset or delay of survey recording relative to time of observation. Investigations into the time window around agitation, as well as enhanced annotation of agitation that marks start and end time points, could provide insight into this. In addition, expanding on the amount of data included here to account for patient heterogeneity (in demographics as well as behavioral phenotype) will increase the ability of personalization in future models.

## Conclusion

In this study, we presented an exploratory analysis of how physiological and movement features, derived from a wearable sensor, relate to agitation and its’ subtypes. We found that there are informative features derived from a wristwatch-like wearable for agitation detection, such as those derived from EDA, ST, HR, and acceleration. Moreover, we observed that the difference in relative response level per feature can indicate different types of agitation, such as verbal or motor agitation. Although population-level trends are mostly consistent across patients, we also noted that model personalization either on a group or individual level would better capture the nuances in the magnitude of responses seen in specific patients. The findings presented here give a fundamental overview of the basic physiology and movement response seen when a person with dementia is agitated. These findings suggest that wearable sensors can provide valuable information about the physiological and behavioral correlates of agitation in dementia, and aid in the development of models for agitation detection.

## Supplementary Material

igae057_suppl_Supplementary_Material

## Data Availability

The data that support the findings of this study are not publicly available due to their containing information that could compromise the privacy of research participants but are available from the authors upon reasonable request. The study was not preregistered.
