# Supplementary material for: Physiological Profiling of Agitation in Dementia: Insights From Wearable Sensor Data
Source: Innov Aging. 2024 Jun 5;8(7):igae057. doi: 10.1093/geroni/igae057 (PMC11227003; doi:10.1093/geroni/igae057)
Supplement: igae057_suppl_Supplementary_Material [file igae057_suppl_supplementary_material.docx]

***Innovation in Aging* Supplementary Material: Davidoff et al. Physiological Profiling of Agitation in Dementia: Insights from Wearable Sensor Data.**

### A-1 Beta coefficients and p-values for all variables and agitation types: (first row each type=coefficient, second row= p-value)

Accelerometer magnitude

|  | Max | Median | Min | Std |
| --- | --- | --- | --- | --- |
| Agitation | **0.305**^**^ | -0.197 | **-0.395**^***^ | **0.455**^***^ |
|  | *0.004* | *0.140* | *0.000* | *0.000* |
| Motor | **0.348**^**^ | **-0.374**^**^ | -**0.393**^***^ | **0.451**^***^ |
|  | *0.002* | *0.009* | *0.001* | *0.000* |
| Verbal | **0.220**^.^ | -0.266 | -**0.305**^*^ | **0.363**^**^ |
|  | *0.096* | *0.107* | *0.025* | *0.004* |

Electrodermal activity (EDA: total, phasic, tonic)

|  | Total | | | | Phasic | | | | Tonic | | | |
| --- | --- | --- | --- | --- | --- | --- | --- | --- | --- | --- | --- | --- |
|  | Max | Median | Min | Std | Max | Median | Min | Std | Max | Median | Min | Std |
| Agitation | 0.085 | 0.052 | 0.066 | 0.119 | 0.165 | **0.255**^*^ | 0.043 | **0.206^.^** | **0.303**^*^ | 0.166 | -0.074 | **0.288**^*^ |
|  | *0.512* | *0.687* | *0.625* | *0.354* | *0.167* | *0.034* | *0.718* | *0.096* | *0.016* | *0.171* | *0.577* | *0.028* |
| Motor | 0.147 | 0.134 | 0.160 | 0.141 | 0.146 | 0.190 | 0.140 | 0.185 | **0.333**^*^ | 0.204 | -0.032 | **0.273**^*^ |
|  | *0.289* | *0.323* | *0.284* | *0.306* | *0.247* | *0.123* | *0.380* | *0.164* | *0.011* | *0.109* | *0.831* | *0.047* |
| Verbal | 0.069 | 0.013 | 0.054 | 0.011 | **0.261^.^** | **0.425**^**^ | -0.057 | **0.336**^*^ | **0.436**^**^ | **0.237^.^** | 0.009 | **0.354**^*^ |
|  | *0.663* | *0.935* | *0.738* | *0.944* | *0.055* | *0.001* | *0.644* | *0.018* | *0.002* | *0.075* | *0.961* | *0.010* |

HR:

|  | Max | Median | Min | Std |
| --- | --- | --- | --- | --- |
| Agitation | 0.148 | **0.537**^.^ | 0.285 | -0.124 |
|  | *0.485* | *0.073* | *0.205* | *0.516* |
| Motor | 0.245 | **0.753**^*^ | 0.304 | -0.091 |
|  | *0.279* | *0.019* | *0.221* | *0.657* |
| Verbal | **0.492**^.^ | 0.481 | 0.319 | 0.213 |
|  | *0.076* | *0.227* | *0.239* | *0.364* |

ST:

|  | Max | Median | Min | Std | Slope |
| --- | --- | --- | --- | --- | --- |
| Agitation | **-0.260**^*^ | **-0.291**^*^ | **-0.236**^*^ | 0.028 | 0.073 |
|  | *0.038* | *0.014* | *0.033* | *0.764* | *0.412* |
| Motor | **-0.282**^*^ | **-0.328**^*^ | **-0.272**^*^ | 0.059 | -0.020 |
|  | *0.043* | *0.011* | *0.025* | *0.547* | *0.832* |
| Verbal | -0.170 | -0.181 | -0.153 | 0.053 | **0.224**^*^ |
|  | *0.269* | *0.213* | *0.270* | *0.644* | *0.046* |

### A-2 Generalized mixed model structure per signal modality:

For accelerometer models:

*Agitation ~ variable + time_group + (1|p_id).*

For EDA and ST models:

*Agitation ~ variable + time_group + average temperature + (1|p_id).*

For HR models:

*Agitation ~ variable + time_group + std. accelerometer magnitude + (1|p_id).*

### A-3 Survey counts by modality and quality

| **# surveys: *1105*** | **# surveys with wearable data present: *792*** | | | |
| --- | --- | --- | --- | --- |
| **Modality** | **EDA** | **HR** | **ST** | **ACC** |
| **# of surveys with high quality** | 444 | 345 | 746 | 792 |
| % of surveys with data (792) where quality = good | 56.06 | 43.56 | 94.19 | 100 |
| **# of surveys with high quality from patients included in modelling** | 442 | 342 | 746 | 792 |
| % of surveys included in modelling out of total surveys (1105) | 40 | 30.95 | 67.51 | 71.67 |

A-3 caption: Survey counts and percentages split by modality and quality. Quality is defined as a combination of the number of data points present per window and the outcome of modality-specific quality indicators. For specific details on the quality per modality, see section ‘Pre-processing and quality control for wristwatch data’. EDA: electrodermal activity. HR: heart rate, ST: skin temperature, ACC: accelerometer.
